# Editorial for the Special Issue on Emerging Memory and Computing Devices in the Era of Intelligent Machines

**DOI:** 10.3390/mi11010073

**Published:** 2020-01-09

**Authors:** Pedram Khalili Amiri

**Affiliations:** Department of Electrical and Computer Engineering, Northwestern University, Evanston, IL 60208, USA; pedram@northwestern.edu

Computing systems are undergoing a transformation from logic-centric toward memory-centric architectures, where overall performance and energy efficiency at the system level are determined by the density, bandwidth, latency, and energy efficiency of the memory, rather than the logic sub-system. This is driven by the requirements of data-intensive applications in artificial intelligence, autonomous systems, and edge computing. We are at an exciting time in the semiconductor industry where several innovative device and technology concepts are being developed to respond to these demands, and capture shares of the fast growing market for AI-related hardware. The collection of articles in this special issue on “Emerging Memory and Computing Devices in the Era of Intelligent Machines” is devoted to highlighting some of the latest advancements in this area, drawing on work on emerging memory devices including magnetic, resistive, and phase change memories, their related circuit and material-level issues, and emerging architectures based on logic-in memory and in-memory computing concepts. A few articles also highlight some of the recent advances in engineering conventional memories—notably Flash and DRAM—to extend and push their performance limits. 

The existing memory hierarchy in electronic systems is characterized by a tradeoff between cost per bit (or, more or less equivalently, bit density per unit area on a chip) and performance (read/write speed). On the slow (highest-density) end is NAND Flash, while the other extreme is (fast but low-density) static random access memory (SRAM), with dynamic RAM (i.e. DRAM) falling in between. Considerable gaps in price per bit and performance exist between NAND and DRAM, and also between DRAM and SRAM. 

Much of the wide-ranging ongoing work on emerging memory devices and architectures can be classified into three categories: (i) Memories that fall between DRAM and SRAM in terms of both bit density and speed, i.e., those that are denser than SRAM but not quite as fast, faster than DRAM but more expensive. Magnetic random access memory is the leading contender in this realm, where both discrete and embedded solutions are of interest. Existing spin-transfer torque magnetic RAM (STT-MRAM) is the state-of-the-art magnetic memory that has received much traction within the industry as an embedded nonvolatile memory (eNVM), with the potential to also replace some embedded SRAM (e.g., L3 or L2 Cache) driving much of the ongoing work to further improve its characteristics. (ii) Memories that are targeted to fill in the large performance and cost gap of DRAM and NAND Flash, also referred to as storage-class memories (SCM). These memories are most often geared toward discrete parts (though specialized embedded applications exist), where a cost penalty compared to NAND Flash is acceptable provided a faster read/write performance is achieved. Examples of these memories are many resistive random access memories (RRAM) and phase-change memories reported to date, among others. (iii) Work that draws on the advances in any of the above memory technologies, but explores unconventional computing approaches, examples being logic-in memory, in-memory computing, neuromorphic computing, and probabilistic computing concepts, among others.

This special issue covers examples of work in all three of these areas: (i) One of the key areas of MRAM research is the exploration of alternative write mechanisms with respect to STT, which is based on driving currents through the memory bit. The goal of these efforts is to achieve better tradeoffs between write speed, bit density, and endurance, while reduction of the write energy is also a possible advantage. An important example is MRAM based on voltage control of magnetic anisotropy (VCMA) [1], which completely departs from the current-controlled mechanism of STT and instead uses electric fields to write information. In “Recent Progress in the Voltage-Controlled Magnetic Anisotropy Effect and the Challenges Faced in Developing Voltage-Torque MRAM”, T. Nozaki et al. [2] present some of their latest results in the development of this type of voltage-controlled MRAM (i.e., VCM). In “Fine-Grained Power Gating Using an MRAM-CMOS Non-Volatile Flip-Flop”, J. Park and Y. Yim [3] explore some of the advantages of MRAM in terms of power management, by taking advantage of its nonvolatility to enable a flip-flop that retains its information without applied voltage. (ii) There are also several examples of RRAM and phase-change memories discussed throughout the selected articles. These range from material- and cell-level studies (X. Lian et al. [4]; Z. Shen et al. [5]; C. Xie et al. [6]; and K. Drake et al. [7]), to the applications of RRAM in processing of biosignals (Y. K. Lee et al. [8]), neural networks (S. Jo et al. [9]; and S. N. Truong [10]), and nonvolatile processors (X. Xue et al. [11]). (iii) Several of the selected articles discuss new computing paradigms that may take advantage of emerging memory devices (G. Santoro et al. [12] and S. Nam et al. [13]), as well as extensions, modifications, or innovations in existing volatile and nonvolatile memory technologies (at both the device and circuit levels), which may add new functionalities or improve their performance for computing applications (H. H. Shin et al. [14]; S. Yang et al. [15]; A. Subbiah and T. Ogunfunmi [16]; H. E. Yantir et al. [17]; and L. Gan et al. [18]). Finally, in “Development of Bioelectronic Devices Using Bionanohybrid Materials for Biocomputation System,” J. Yoon et al. [19] review their recent progress in the development of biocompatible memory and computing devices.

The selected papers cover a broad range of research and development activities related to emerging memory devices and computing paradigms. It is hoped that this selection of articles will serve as a resource for researchers in academia and industry, practicing engineers, and students, both as a window into some of the recent advances in emerging memory technologies, as well as to stimulate interest in potential new directions for research.
